# Migration, Knowledge Transfer, and the Emergence of Australian Post-War Skiing: The Story of Charles William Anton

**DOI:** 10.1080/09523367.2017.1313234

**Published:** 2016-11-01

**Authors:** Philipp Strobl

**Affiliations:** ^a^ Faculty of Health, Arts and Design, Swinburne University of Technology, Hawthorn, Australia

**Keywords:** Skiing, knowledge transfer, refugees, Alpine Club, ski touring, history of migration

## Abstract

Skiing underwent substantial changes during the post-war years when the sport turned into a multi-billion dollar industry and a leisure activity for the masses. Despite its global nature and popularity, skiing in academic writing has not gained much recognition. This paper explores the role of knowledge transfer during the pioneering phase of post-war skiing in Australia. It describes the life of Charles William Anton, an Austrian refugee from the *Anschluss* who migrated to Sydney and subsequently became one of the founding fathers of Australian post-war skiing. The following pages show the multi-layered nature of skiing as a global sport by exemplifying how ideas spread from pre-war Europe to post-war Australia. The paper will also provide a case study about refugee knowledge transfer and the ‘productive process of absorption, adoption or rejection of knowledge’ that takes place once an idea has been introduced into a new environment.

The difficulties of building in such a remote and inaccessible spot are forbidding. Experts said it couldn’t be done. One man thought differently. He was Mr. Charles Anton.^1^

Australian skiing underwent substantial changes during the post-war years. It changed from being an elitist niche sport into a multi-billion dollar industry and a leisure activity for many more people. The 1950s and 1960s brought intensive investment in ski resorts and an unprecedented hotel-building-boom. It was a time ‘when the sport began to hit the mass market’.^2^ Ideas, trends, and knowledge transferred by migrants played an important role in those developments, particularly in Australia where tens of thousands of skilled European refugees arrived between the 1930s and 1960s. Many of them had practiced skiing as a leisure activity in their former homelands – some were even professional skiers.^3^

This paper explores the role of knowledge transfer during the pioneering phase of post-war skiing in Australia. Although a global phenomenon that ‘has undergone so many improvements and almost metamorphoses’^4^ while spreading throughout the world, skiing, in academic writing, has not ‘gained the recognition accorded to other sports’.^5^ There is very little research on knowledge transfer processes during the early phase of post-war skiing and only a handful of historians have dedicated their work to the analysis of skiing as a global phenomenon.^6^ As far as Australia is concerned, the history of skiing is mainly local history. Subsequently, the sport’s past is described either on a national,^7^ or a regional level.^8^ Knowledge transfer processes, in general, have been thoroughly studied in different academic disciplines. Mitchell G. Ash,^9^ and Johannes Paulmann,^10^ provide essential remarks on the historical study of knowledge transfer and intercultural transfer processes. In more recent years, Veronika Lipphardt and David Ludwig have added their thoughts on the subject.^11^

This paper contributes to the existing scholarship in two ways: Initially, it will show the multi-layered nature of skiing as a global sport by illustrating how ideas spread from pre-war Europe to post-war Australia. Secondly, it will provide a case study about refugee knowledge transfer and the ‘productive process of absorption, adoption or rejection of knowledge’^12^ that takes place once an idea had been introduced into a new environment.^13^ Thus, the paper shows how knowledge was accumulated, imported and adopted by one specific ‘social agent’.^14^ It discusses the life of Charles William Anton, a Viennese ski touring enthusiast who fled to Sydney after the *Anschluss* and subsequently became one of the pioneers of Australian post-war skiing. It explores how he gained knowledge about ski touring and the skiing infrastructure in Austria (knowledge accumulation), how he raised different businesses in his new homeland, and how ideas and know-how gained in Austria influenced his work (knowledge import). The framework and preconditions that allowed him to make a long-lasting impact on Australian snow field developments are also of particular interest here. The paper analyzes how he translated and adapted knowledge gained in Austria to his new environment thereby transforming ‘him, the receiving context, as well the knowledge itself’ (knowledge adoption). ^15^ It argues that his knowledge of Alpine Clubs and organizations in Austria initially enabled him to create the largest ski club in Australia whose structures and organization differed greatly from already existing Australian clubs. It further argues that he successfully adapted his initial plans to new market needs, not least because he kept himself well informed about overseas skiing trends and developments. The creation of networks is essential for the establishment of new businesses, particularly for migrants entering a new and initially unfamiliar environment. The paper, finally, will explore how he formed and maintained extensive networks, how his emigration experience supported this process, and how Charles Anton used Austrian elements to launch successful marketing campaigns to promote his plans and skiing in Australia.

## ‘Every Night in a Different Alpine *Hütte*’: Charles Anton’s Youth in Austria

Charles William Anton was born Karl Anton Schwarz in Vienna on 23 November 1916. The second child of the timber merchant Alois Schwarz and his wife Stella, he grew up in the Viennese suburb of Grinzing.^16^ Like many other Viennese Jews, ^17^ the Schwarz family were assimilated Jews. Alois Schwarz was born in 1871 in the Austrian province of Galicia. After moving to Vienna, he had left the Jewish faith and was baptized a Catholic.^18^ His mother’s Jewish family had moved from Slovakia to Vienna around the turn of the twentieth century.^19^ After elementary school, Karl Anton attended the Technologisches Gewerbemuseum,^20^ a technical high school known for its excellent educational standards. He obtained his high school leaving certificate in 1935.^21^ After graduating, he worked for the Austrian branch of the British Sun Insurance Company and was among the few young Austrians to get a job in an international company. His job allowed him to gain a good command of the English language, far beyond basic school English. Many Australians were later impressed by his excellent language skills.^22^ In April 1938, Sun Insurance Company came to the notice of the Nazis, for at least one or their proxies was Jewish and had to resign shortly after the *Anschluss* when Jews were forced out of influential positions in public and private companies.^23^

Already during his youth, Anton discovered the Austrian mountains around Vienna and very soon had become ‘enthused with what ski touring had to offer’.^24^ The sport was very popular at that time,^25^ especially as a leisure activity for city people. Since the 1870s, the Austrian Alpenverein (Alpine Club) had built up a dense network of *Hütten* – ‘simple huts offering security and shelter’^26^ – across the Alps and thus offered an excellent infrastructure for those interested in mountaineering.^27^ Since about 1900, mountaineers began to tour the Alps on skis during the winter. Consequently, the Alpenverein lured ever more enthusiasts and established a ski-sub-organization in 1906.^28^ In order to realize its ambitious hut-building-schemes, the governing body of the Alpenverein transferred single projects to independent sections whose members raised most of the costs by selling shares.^29^ Discounts on overnight stays encouraged members to visit other huts of the organization.^30^ Charles made extensive use of the Alpenverein’s infrastructure and acquired knowledge of the organization’s structures and the value of a dense network of shelter huts in alpine areas.^31^ He learned where fitting locations for huts could be found, how they could look and how they were maintained. One of his acquaintances later wrote that ‘Charles and his friends – with a pack on their backs – would finish up almost every night in a different alpine *Hütte*’.^32^

The interwar period brought ‘the arrival of mass tourism in the Alps, however, contradicted the narrative preferred by the Alpenverein’.^33^ Ski lifts and new hotels were built across the Alps during the 1920s and 1930s.^34^ Mountaineers claimed that ‘profit-motivated investors were ruining the landscape as they commercialize it’.^35^ Their reactions to the development of an early tourism industry showed ‘a great deal of resentment and fuelled an increasingly elitist attitude’.^36^ Many mountaineers tried to segregate from the masses of tourists that ‘were brought to the mountain summits en masse by lifts’.^37^ They rejected mass tourism and the exploitation of the mountain landscape and regarded themselves as an elitist circle, as the only ones who had the right to explore the mountains. Socialized in that environment, young Charles might have been influenced by that attitude, as some of his later statements indicate.^38^

## ‘Seeing the Writing on the Wall’: *Anschluss* and Escape to Australia

The proclamation of the so-called May constitution of 1934 brought an early end to Austria’s democratic development. The republic turned into an authoritarian regime repressing its enemies on both sides of the political spectrum.^39^ Tensions between the Catholic Austrian and the National Socialist German regime increased rapidly during the mid-1930s, pushing the Austrian Government towards military armament.^40^ One of the measures taken in 1936 was the introduction of a compulsory military service. In September 1937, Anton was drafted.^41^

On 11 March 1938, National Socialists seized power in Austria. Four days later, Adolf Hitler announced to a crowd of 200,000 jubilant Viennese that ‘Austria had joined the German *Reich*’.^42^ The vast majority of Austrians welcomed the *Anschluss* with ‘unbridled enthusiasm’ and tried to take advantage of the new situation.^43^ Jews were soon removed from all state positions,^44^ including the army. Many people, like the Catholic Schwarz family, who did not consider themselves Jewish, were made Jewish by law. Jews were no longer regarded as citizens and thus subject to a series of discriminatory and later deadly measures, rigorously defined in the Nuremberg Laws. Anton was dismissed from the army as early as March 1938.^45^ He had refused to take the compulsory ‘oath of allegiance to Hitler’,^46^ and shared the fate of ‘thousands of Austrian officials’, who consequently lost their jobs.^47^ Anton, and his family ‘saw the writing on the wall’, as one of his friends remembered.^48^ When they heard about Austrian army officers who had been imprisoned in concentration camps, because of being Jewish and consequently ‘were kicked to death’, emigration became inevitable.^49^ Very soon after his release from the army, he escaped Austria. He found a job with the Australian Jewish Insurance broker company Bennie S. Cohen & Son Pty Ltd,^50^ which entitled him to one of the rare visas for Australia. He spent his last months in Europe travelling to different countries and rigorously preparing his and his parents’ escape.^51^ Thanks to his efforts and foresight, his parents had opened up a bank account in Switzerland and were able to secure some of their financial assets.

Anton arrived in Sydney on 6 December 1938.^52^ During the following months, the 22-year-old quickly settled into his new environment. He successfully commenced his job and made friends in Australia. Only two months after his arrival, he changed his name by deed poll to Charles William Anton.^53^ His assimilation was smooth and quick, as many of his friends later remembered. Described as ‘fairly anglicized’,^54^ he frequently impressed Australians with ‘fancy words’, intending to show that he had mastered the language.^55^ Thus, he differed from many who escaped Austria during those days. The majority had severe difficulties finding a job and tended to associate with other refugees, because of initial language, cultural and financial difficulties.^56^

Like many other Austrian refugees, Anton wanted to join the Australian army after the outbreak of the war. ‘I sincerely hope that you will give me the chance to do my bit in this war which after all is as much our war as it is yours’, he wrote to a recruiting officer in 1940.^57^ In March 1942, he was drafted to the 3rd employment company in New South Wales, a new unarmed military body that was in part or whole made up of refugees who were classified as ‘enemy aliens’.^58^ A few days later he married his first wife Betty Estelle Caldwell.^59^ After the outbreak of the war, resentments towards ‘enemy aliens’ increased rapidly. There was an emotional public discourse around the question, of whether foreigners should be allowed to serve in the army. In November 1942, Private Anton published an article in the *Sydney Morning Herald* stating that refugees escaping from Hitler’s oppression view the discourse ‘with sorrow and consternation’. He further added: ‘They [foreign soldiers in the employment companies] are all volunteers. They have taken the oath of allegiance to the King and are sharing the risks, sacrifices, and privileges of all Australian soldiers’.^60^ Refugees had a long struggle for official recognition of their status. For the first four war years, Australian authorities did not distinguish German- or Austrian-born refugees from other ‘enemy aliens’.^61^ Only in 1943, Arthur Calwell, chair of the Aliens Classifications and Advisory Committee, officially stated that ‘it was both absurd and unjust to treat refugees from Nazi-Germany as enemy aliens’.^62^ Consequently, they were able to apply for reclassification as ‘refugee aliens’. Anton’s status was changed on 1 February 1944.^63^ Seven days later his daughter Marina Louise was born.^64^ Anton was naturalized four months later (June 1944),^65^ and finally transferred to the Australian Imperial Forces in July 1945.^66^

## ‘Snow in Australia? That’s News to Me’: Exploring the Australian Alps and the Origins of the Ski Tourers Association

Skiing in Australia takes place in three active skiing states (New South Wales, Victoria, and Tasmania), as well as the Australian Capital Territory and has a long tradition: During the second half of the nineteenth century, Norwegian miners were the first to bring the idea of skiing to the gold rush mining town of Kiandra in New South Wales.^67^ Shortly afterwards, migrant miners introduced skiing to Victoria. The state of Tasmania was the last to join Australia’s skiing states during the 1920s when the Austrian immigrant Gustav Weindorfer introduced the sport.^68^ Skiing became popular among those who could afford the expensive equipment and the journey to the mountains. A handful of huts and hotels for tourists were established in the Victorian and New South Wales Alps during the late 1890s and early 1900s. Some more accommodation was built during the interwar period. The supply, however, could not keep up with the demand. From the late nineteenth century onwards, Australia’s first ski clubs were formed: ‘Kiandra Pioneer Club’ was the country’s oldest ski club. By 1900, its membership exceeded 100.^69^ More clubs were soon to follow: Between 1909 and 1944, 11 ski clubs were founded.^70^ Some of them already built up their own club lodges. The most prominent were the Kosciusko Alpine Club (1909), the Ski Club of Australia (1920) and the Ski Club of Victoria (1924).

At the close of the hostilities, the Allied powers held an ‘Inter-allied Ski race’ in New South Wales. Servicemen from different countries participated in this major event.^71^ Anton joined the Australian team and experienced his ‘first taste of Australian skiing’.^72^ After his demobilization in December 1945,^73^ he continued to work as an insurance broker and consequently established a successful business in Sydney. The races, however, had reawakened his passion for ski touring.^74^ In spring 1946, he started to discover the main range of the Australian Alps and found, ‘superb runs, comparable to some of the best in the European Alps’,^75^ and ‘began to dream of opening up it [that country] for others’.^76^ In March 1948, he divorced his wife Betty Estelle. He had met the journalist Margaret Foster on a ski trip and they married in July 1948.^77^ Margaret was also fascinated by the sport and supported his ideas.^78^

Up until the 1940s, skiing in Australia was widely regarded as a sport of the so-called establishment.^79^ Because of the very low number of skiers, infrastructure was very underdeveloped and many criticized the ‘backward state’ of skiing in the Australian Alps.^80^ Anton had recognized that there was ‘a demand for touring lodges in the heart of the Alps’,^81^ and developed plans to found a mountaineer organization similar to the Austrian Alpenverein. The realization of his plans took years of preparation: He finally convinced the state park authority to provide land for a hut and approached different ski clubs as well as the ‘members of the skiing fraternity’.^82^ ‘He had a gift for organizing people and it was that gift that underpinned the success of the STA’, a sports journalist later recalled.^83^ When Anton held the first meeting to discuss his ideas in October 1950, he had gathered the major figures of the New South Wales skiing community as well as high-ranking ‘representatives of the State Park Trust, the Government Tourist Bureau, and the Ski Council of New South Wales’.^84^ According to the *Australian Ski Yearbook*,

Support for the project was unanimous and … the meeting felt confident that it could look forward to practical assistance from the trust … Finance was arranged … and … the site has been tentatively chosen … During this meeting, [the] Ski Tourers Association [STA] came into being.^85^

When he founded the STA, Anton used his knowledge of the structures of the Alpenverein to establish an inclusive organization that differed greatly from already existing exclusive Australian ski clubs. Following the pattern of the Alpenverein,^86^ he established a co-operative concept, whereby autonomous Club projects affiliated to form an association with a common constitution.^87^ Sixty members initially joined the Association’s first hut project at Lake Albina. Many of them were migrants themselves.^88^ Here again, Anton showed his ability to create business networks that helped him ‘[translate] his plans into action’.^89^ He approached local entrepreneurs who offered tools and supplies, such as a bulldozer, timber, snowmobiles, packhorses, and so on,^90^ and gained support from volunteers. Anton recognized the value of people with alpine experience and regularly visited the construction sites of the Snowy Mountain scheme, a hydroelectricity and irrigation complex in the Snowy Mountains that was mainly built by European immigrants,^91^ in order to hire skilled people. The Czech migrant Tony Sponar gives a colourful description of how Anton had recruited him for the Lake Albina project:

On our first weekend at the Chalet, a short, noisy man appeared. In no time, he had introduced himself as Charles Anton and just as fast he had told me that I should be the one to help him with his current project. He had organized a few other volunteers along with two of the Kosciusko State Park Trust’s Land Rovers.^92^

The building process was accompanied by a media campaign, boosted by Anton’s second wife Margaret. Newspapers announced the hut as ‘Australia’s highest habitation’,^93^ as a ‘favorite fantasy of main range skiers’,^94^ and as a place where the ‘ski runs are best and the snow lasts longest’.^95^ From the very beginning, Anton knew about the importance of public events to promote his plans. When the hut was completed, STA threw a party at the Sydney County Council,^96^ and invited many high-ranking officials and representatives of other ski clubs.^97^ This was the beginning of a series of glamorous fundraising and advertising events to advance his aims and promote ski touring. A STA member later remembered: ‘it was due to his [Anton’s] flair for publicity that the Australian public was made aware of the tremendous resources of our snowfields’.^98^

Anton was always looking for new ideas. His passion brought him to the mountains even during the hot Australian summers. As he described in the *Australian Ski Yearbook*, he discovered already in 1946 ‘that there was snow – and we could ski on it – … in the summer’.^99^ From that time, he frequently visited the Australian Alps also during the summer time and developed the idea of organizing summer ski races.^100^ Only a year after the STA was founded, Anton established summer ski races with Lake Albina hut as headquarters for the race committee.^101^ The races were held on a drift of snow and brought together competitors from different countries.^102^ Held annually for many years, they attracted ever ‘larger numbers of visitors’ and thereby succeeded in promoting the STA as well as skiing.^103^ Anton was convinced that the ‘freakish nature’ of the races would ‘produce a welter of favorable publicity’.^104^ In 1953, he established the Golden Eagle Run, a high-speed downhill run that was expected to be the fastest course in Australia. Here again, knowledge from Austria was used to support the project’s success. Anton, as he later stated, was inspired by ‘famous international courses including the *Chamois Run* in Kitzbühel and the *Kandahar Run* in St. Anton’.^105^ The former Czech olympic racer and St Anton ski instructor Tony Sponar helped him to develop the plans and set the course according to European Alpine standards.

Lake Albina Hut was a milestone in Australia’s skiing history. After the lodge was finished, it was possible ‘for even greater numbers of people to ski the western part of the Main Range in comparative safety’.^106^ Anton’s ‘enthusiasm’ and commitment, combined with active marketing and an inclusive policy turned the STA very soon into the largest ski club in Australia.^107^ Lake Albina hut was the ‘first completed club lodge in Kosciusko state park’.^108^ Very soon, a number of clubs followed the successful example and constructed their own lodges. Through winter 1951, 180 skiers joined the STA. The lodge was such a success that Anton, ‘who was not prepared to sit back, even for a short time’,^109^ launched another hut building project. As early as November 1951, the STA chose a site for a new venture in Kunama Valley. Work commenced in February 1952, however thunderstorms caused severe damage and deferred its completion. Opened in 1953, ‘Kunama Huette’ was named after ‘Kunama’, an Aboriginal word for snow.^110^ Anton had a clear vision of how it should look like. He wanted the hut to be like one of the Alpenverein *Hütten* he knew from Austria. Hence Austrian influences were visible almost everywhere. There was a long struggle among STA members about what the lodge should look like. At one meeting Anton finally succeeded with his ideas. After displaying a cuckoo clock, he said: ‘Why don’t we make it like this’.^111^ The *Hütte* was equipped with ‘all modern comforts … and was built and furnished in a Tyrolean style’.^112^ STA even hired an ‘area manager’ with an Austrian family background.

Why did Anton cling to so many Austrian cultural elements? His friends confirm that he tried everything to be an assimilated new Australian citizen. He also did not seem to have many sentimental feelings towards his homeland. It is most likely that he used Austrian elements to stand out from others and to create publicity: Austria was very fashionable among Australian skiers at that time given the fact that much of Australia’s ski know-how derived from Austria. Once World War II was over, more Australians ‘began taking overseas holidays and returning with glowing reports of ski lifts’ in Austria.^113^ Australian affinity towards Austrian skiing had a long tradition. The first Austrian skiing instructors arrived as early as the 1930s. Their arrival ‘established the pattern of importing skiing instructors from Austria and ensured that the Austrian influence on Australian skiing would be a lasting one’.^114^ ‘The Austrian experts were hailed; they were the rock stars of the snow, celebrities in the village, performers on the mountain and keepers of the [ski] knowledge’, a sports journalist later recalled.^115^ Anton’s marketing plans worked out very well: The 10-bed lodge was an ‘outstanding success’,^116^ and the media responded positively. The *Hütte* was announced as a ‘Tyrolean-style hut at Kosciusko’,^117^ or as a ‘copy of a Tyrolean chalet’.^118^ The project was the one ‘dearest to Anton’s heart’ as Wendy Cross, long-term editor of the *Australian Ski Yearbook* put it.^119^

At the time when ‘Kunama Huette’ was built, STA, ‘in accordance with modern trends’, decided to build a ski tow close to the hut. The first ski-lift in Australia was built in Victoria in 1937,^120^ and up until the early 1950s there were very few ski tows in the Australian Alps. Skiers either had to walk uphill or had to be pulled by a snowmobile. Already during the 1920s, alpine resorts in Europe, the United States and Canada had introduced cable cars and ski tows. On one of his tours through the Austrian Alps, Anton might have seen the two modern cable cars in Innsbruck which were built in 1928^121^ and had lured more than 311,000 visitors annually into the mountains around the city.^122^ He also might have encountered the rope tows of the skiing resort of Zurs in Vorarlberg or St Anton in Tyrol that already had recognized the advantages of a fast uphill transportation allowing skiers to improve their downhill race techniques. During the 1950s, many Australian skiers called for new uphill transportations. A 1950 *Australian Ski Yearbook* article summed up these demands: ‘All … skiers, especially those who have been fortunate enough to visit skiing resorts of America and Europe, are agreed that ... all slopes worthy of the title of ski-run should be equipped with ski-tows’.^123^ Anton helped meet those demands by building the ‘highest ski tow in Australia’^124^ in 1954 at Mount Northcote next to ‘Kunama Huette’. The ski tow project was new to him. In one of his letters to the STA members, he described it as ‘an experiment which might prove a boom to your own skiing and to main range developments generally’.^125^ After start-up difficulties, the tow became increasingly popular, not least because it was used as the means of transportation for the popular ‘eagle runs’ and as a training facility for the Australian Olympic Ski team.^126^ As early as 1954, STA decided to extend the Tow House to provide overnight accommodation for skiers.^127^

Austria retained a special position in the heart of ‘fairly anglicized’ Charles Anton.^128^ His life shows an interesting contradiction between his pride in being assimilated and his embrace of Austrian cultural paraphernalia. On the one hand, Anton never spoke much to friends about his past in Austria.^129^ He was proud of his British accent and his active English vocabulary. On the other hand, Anton’s affinity for his old *Heimat* influenced his private life. He and his second wife Margaret furnished their entire flat in the Sydney suburb of Randwick as an ‘Austrian alpine peasant’s home’.^130^ Since traditional Austrian furniture was unobtainable, the Antons spent months creating their own Tyrolean style furniture. Chairs, tables, cupboards, beds, wooden ceilings, everything was made of Australian raw materials with the ‘help of a young apprentice cabinet maker’.^131^ Miniature paintings of Tyrolean landscapes and a cuckoo clock on the wall accomplished their bit of rural Austria in suburban Sydney. Here again, Anton’s passion for the ‘extraordinary’ and his desire to stand apart from others could offer an explanation. Described as ‘vain’ by friends,^132^ he always wanted to stand out. He used special fonts on his German-keyboard typewriter,^133^ and drove around Sydney ‘in a gleaming black Mercedes coupe with a flag flying from the bonnet bearing the insignia President of the Ski Tourers Association’.^134^ Being the only person in post-war Sydney with a Tyrolean-style apartment certainly was a unique feature, even attracting newspaper coverage.^135^

Lake Albina Hut and ‘Kunama Huette’ constituted the success of the STA. Both lodges were described as the ‘highest buildings in Australia’ at that time and ‘the only club huts on the main range, where the snows last longest’.^136^ Soon another hut project followed. In 1956 a proud Anton wrote: ‘we are well on the way to achieving our aim of a chain of lodges’. At the onset of the 1956 winter season, STA, whose total membership exceeded 500, already had built four lodges, providing more than 40 beds in different locations of the Alpine main range.^137^

## ‘New Times Arriving!’: Club Lodges and Thredbo

STA suffered tragic setbacks in 1956. Within three weeks both an avalanche destroyed ‘Kunama Huette’, killing one of its occupants, and a fire devastated the Northcote Tow House.^138^ ‘In the short span of three weeks, the results of the four years’ hard work have been completely wiped out’, an exhausted Anton complained.^139^ After the disaster, Kosciusko State Park introduced new environmental and safety policies that made rebuilding Kunama Huette impossible and ‘without the *huette*, there was no point in rebuilding the tow’.^140^ The disaster, new regulations, as well as improvements in skiing in general, forced Anton to reconsider his initial plans. When he founded STA, he was influenced by the Austrian Interwar mountaineer attitude of a rejection of mass tourism. Initially, he wanted to make the Australian Alps accessible to people who seemed physically fit and able to handle the dangers of the mountains, and did not consider opening up the Alps to mass tourism. ‘I want to keep it for the [mountaineer] elite’,^141^ he told his friend and later business partner Tony Sponar during an argument over the location of Mount Northcote Ski Tow.

From the mid-1950s, skiing in Australia was about to change. Influenced by a post-war ski boom in Europe and the United States, alpine skiing turned from a mere leisure activity of a few mountaineering alpinists into a mass ‘industry’.^142^ New alpine resorts mushroomed across the European Alps and Austrian resorts substantially improved their infrastructure, not least with the help of significant Marshall Plan funds.^143^ Ski weekends and annual weeklong ski vacations had become ‘de rigueur for much of the middle class’.^144^ European developments boosted the plans of Australian ski enthusiasts and snowfield developers. Within a few years, equipment had improved towards a better control of downhill running, ski tows had started to become popular and private club lodges had mushroomed along the Australian Alps. Commercial hotels were also being erected to offer meals and evening recreation.^145^ The Australian Alps were no longer underdeveloped terrain, and Anton’s idea needed to adapt to the new conditions. The rapid increase in the number of lodges forced the State Park Trust to establish controls over the spread of development and it took steps to concentrate development into designated resort areas.^146^

Eighteen years after his arrival in Australia, Charles Anton had become an established name in the Australian skiing community. He was president and founder of a major Australian ski club and a member of the Ski Council of New South Wales, the State controlling body of the Australian National Ski Federation.^147^ People knew he had the experience, know-how, and contacts to realize complex projects. He had built up a valuable network of contacts. He had dealt with different ski clubs, had approached local authorities, had maintained excellent contacts with the Kosciusko State Park Trust and had hired European migrants for his hut-building projects.

One of them was the Czech migrant Tony Sponar, who had worked in the Tyrolean ski resort of St Anton. Sponar came to Australia with an excellent knowledge of how a major ski resort works and was fascinated by the idea of opening up a glamorous, European-style resort in Australia.^148^ During the early 1950s, he had approached Anton several times about his idea, however, could not stimulate his interests. In 1955, finally, Anton might have recognized the changing conditions and the need for the adoption of his plans. ‘I had lunch with Charles Anton who was accompanied by Geoffrey Higgins. They have been thinking over your Thredbo proposal and have become convinced, as I have, that it has very many attractive aspects in it’, wrote one of Anton’s later business partners to Sponar in 1955.^149^ Those words marked the beginning of ‘Australia’s best and most international ski resort’.^150^ Inspired by Sponar’s idea, Anton began to dream about opening up a ‘ski carousel á la Kitzbühel’, as he later stated.^151^ Sponar was glad to have Anton aboard since he knew about his organization skills and business contacts. ‘I enthusiastically embraced Anton’s participation. I did not know the other person named, but being in Anton’s company was good enough for me’,^152^ Sponar later recalled, thus indicating Anton’s excellent reputation.

Sponar had the vision, insights, and knowledge about European ski resorts, however, he lacked the networks and skills of bringing together the ‘right kind of people’ to successfully realize his concepts. That was Anton’s domain. After being convinced of the ‘attractive aspects of the project’, Anton started contacting people who might be able to support the project ‘with advice and money’.^153^ ‘Hyperactive as a bumble bee in a bottle’,^154^ he spoke with various possible investors and experts from Australia, Austria, and the United States.^155^ Finally, in May 1955, a syndicate was formed to develop Thredbo.^156^ To meet their financial needs, Anton brought in the entrepreneur and philanthropist Thyne Reid^157^ who contributed £6,000 and ‘kicked off the project’.^158^ In 1957, ‘Kosciusko Thredbo Village Ltd’ obtained a 99 years lease for 27 hectares of land.^159^ The lease included a franchise over the surrounding 1,849 hectares with exclusive rights to operate ski lifts, ski schools, service station and liquor outlets^160^ and was linked to the completion of an 80-bed hotel as well as a double chairlift within the first five years.^161^ This obligation put the syndicate under financial pressure. Unable to cover the costs of building up the resort out of its own pocket, the syndicate decided to form a publicly listed company and subleased sites for lodges to different ski clubs. The initial public response was poor and the undertaking seemed to be on the brink of financial disaster. In that situation, Reid poured in more of his own money and underwrote a loan.^162^ Anton also raised a further £1,000. Together, they financed ‘an access road from the Alpine way, as well as … a fully equipped commercial lodge, providing meals and accommodation’.^163^

At that time Anton had already reconsidered his initial STA-plans. New regulations had complicated his vision of a chain of touring lodges across the Australian Alps and skiing was about to become a leisure activity for the masses. Instead of building touring and shelter huts in exposed positions (as Australian duplicates of what Chares had seen in Austria), STA now focused on ‘accommodation lodges in promising ski resorts’.^164^ By adapting an idea, originally developed for the European Alps, Anton created new organizational patterns and thus opened the Club to the growing numbers of Australian post-war leisure skiers. The initial structure of the STA as a superior organizing body as well as its inclusive direction remained unchanged, while the nature of the association’s huts and thereby also its clientele altered.

Between 1958 and 1959, STA built two lodges in Thredbo offering accommodation for 38 skiers. A proud Anton described one of them, ‘Kareela Huette’, as a ‘worthy successor of the avalanche destroyed Kunama Huette’.^165^ Here again, Austrian influences were striking. Anton had hired the Austrian architect Otto Ernegg to build the hut in a contemporary Alpine style. A glittering ‘Roof-Raising Party’ (another Austrian tradition), with more than 100 prominent guests,^166^ helped publicize the hut. Because of its exquisite location on top of the Thredbo chairlift, Anton used the lodge for different public events. ‘Out of all proportions to its size’, he later wrote, ‘Kareela provided the setting of … widely publicized events … and thus helped to popularize Thredbo and skiing in general’.^167^ Despite initial kick-off problems and difficulties among some members of the syndicate, Thredbo ‘seems to be growing faster than any other ski area in Australia’.^168^ Two years after the company had obtained the lease, Thredbo village comprised 27 lodges and hotels and provided more than 400 beds.^169^ After another two years, the resort offered accommodation for more than 1,000 people and the value of private construction in the village was estimated at £1 million.^170^ Further know-how from Austria was imported in 1958/59 when Thredbo established its first ski-school, staffed by two Austrian instructors. With respect to the employment of new instructors, ‘Charles naturally turned towards Austria’, as one of his friends described.^171^

Anton did much to publicize Thredbo. He knew about the promotional effects of major skiing events and brought the State Ski Championship to Thredbo in 1958. A newly established STA-lodge was used as headquarters for the race committee.^172^ He also knew ‘about the value of including dignitaries in the resort’s activities’.^173^ When the members of the syndicate were searching for the ideal location for the villages’ main hotel, Anton ‘as it was in his nature, took himself to organize special occasions for people of influence to express their opinions about their preferences of the site’, as Tony Sponar described it.^174^ Geoffrey Hughes, another of his Thredbo business partners, mentioned in his biography: ‘In those days, Charles Anton was able to get publicity for Thredbo through stunts, such as having sundry clerics come to Bless the Snow, with vestments flapping in the stiff breezes at the top of the Thredbo Chairlift’.^175^

During 1960 and 1961, the company’s financial resources could not cope with the needs of the fast growing resort.^176^ Kosciusko Thredbo Village Ltd had difficulties raising enough money for a major state-of-the-art hotel with *ensuite* facilities^177^ and realized that only sufficient financial means would allow for further growth. Thus, in 1961, they ‘discretely put it [the company] on the market’,^178^ and finally agreed to a takeover by a major property and infrastructure company. Satisfied with the outcome^179^ Anton stated in an interview in 1961: ‘Lend Lease [the buyer] has the means and the organizing ability to make Thredbo valley … the center of the most highly concentrated winter and summer tourist area in Australia’.^180^

## ‘Cashing in the Trade’: The Australian Alpine Club, Ski Resorts, and Expansion

The takeover put an end to Anton’s direct involvement in Australia’s main ski resort. It coincided with a new period in his life: After divorcing his second wife, he married Jutta Margaret Eva Olivier, a 25-years-old post-war immigrant from Germany.^181^ The money acquired in his Thredbo venture allowed Anton to fulfil a personal dream. In late 1962, he took a vacation to Austria where he visited modern ski resorts and made contacts with Austrian skiers and representatives of the Austrian skiing community.^182^ In addition to his ski projects, Anton always had maintained his insurance business. He was able to connect his business with the needs of an emerging Australian skiing industry and a nationwide Alpine ski club and attracted many clients from the ski community.^183^ A friend of his later described Anton’s ‘tireless commitment to working 15 hours or more per day in order to come along with the high demands of two full-time jobs’.^184^

Thanks to its adapted business concept, STA was able to respond to the needs of the fast growing group of Australian middle-class leisure skiers and experienced a period of rapid growth. In 1963, Anton opened up a lodge in the booming resort area of Perisher Valley, where his ‘original Thredbo aim of opening up a ski carousel or circus a la Kitzbuhel had already been realized’.^185^ Austrian elements again helped him promote the new lodge. He launched a traditional Austrian ‘Glühweinparty’ at a famous Sydney Steak House and praised the excellent location of the ‘European Style Alpine Hut’.^186^

As one of the directors of Thredbo, Anton had frequently visited other resorts and became aware of the potential of other Australian ski regions. Victoria had particularly attracted his interest.^187^ ‘Victoria provided more opportunities for ski club developments than did New South Wales’, he wrote during that time.^188^ He frequently travelled to Melbourne and the ski resort of Falls Creek, thus making contacts with Victorian skiers.^189^ In late 1962, while ‘Perisher Huette’ was built, STA launched its first venture in Victoria. Anton leased a site at the ski resort in Falls Creek, which he described as ‘the most over lifted area considering the numbers of beds’. ‘It has a particularly well-organized ski school, staffed by top-notch Austrian instructors’, he enthusiastically announced.^190^ Anton began to push the project. ‘The early morning phone calls, the letters typed while he was traveling, and the flying visits to Melbourne … really kept us on our toes’, described one of his business partners.^191^ Anton again drew on already established Austrian cultural elements to advertize the project: A lodge launching ‘Glühweinparty’ was held in a Melbourne restaurant, including a traditional Austrian harp and zither combo.^192^

With the shift of interests that had taken place in the STA and its expansion towards ski resorts in other Australian states, the name ‘Ski Tourers Association’ did not seem appropriate any longer. In late 1962, Anton unilaterally decided to establish the organization’s ‘national significance by changing “STA” into “Australian Alpine Club”’ (AAC) thus again drawing back to major European Alpine Clubs such as the *Alpenverein*.^193^ How much he was influenced by Austrian structures can be seen in one of his statements before his departure to Europe, when he told a close friend that the title ‘President of the Australian Alpine Club’ would mean much more in Austria than ‘President of the Ski Tourers Association’.^194^

Skiing turned ‘into a global mark of middle-class status and a motor of economic development during the 1950s and 1960s’,^195^ and Anton, who had recognized that development early on, jumped on that bandwagon by adapting his plans and building up commercial resort huts. After 20 years in Australia, the Austrian interwar mountaineer Karl Anton Schwarz had finally given up his reluctance towards mass tourism and began to see skiing as an ‘important industry’.^196^ He even focused his attentions on the expansion of Australian commercial skiing facilities into new markets. In a speech at the National Tourist Association conference in November 1961, he daydreamed: ‘with Australian mountain facilities rapidly approximating those of the United States, the time is quickly approaching when, in view of our reversed seasons, we will be able to attract North American skiing enthusiasts in considerable numbers’,^197^ and that Australian ski resorts should focus on the 10 million North American skiers and should ‘cash in on that trade by providing first-class facilities’.^198^

During the following years, he continued advertizing skiing and Australian resorts. In 1964, he relocated his summer races to Thredbo making them a big spectacle with more than 1,000 spectators, ‘who rode up the chairlift from the village. Many dressed in colorful summer clothing’.^199^ Two years later, he organized a Ski Cup in Thredbo and secured a visit of ‘the best skiers’ of the Austrian Ski Team.^200^ The race was accompanied by a massive media response and was intended to ‘raise the standard of Australian skiing by giving local skiers valuable racing experiences against the world’s leading ski champions’ as well as to increase the popularity of the sport in general.^201^ Probably inspired by his trip to Austria, he introduced a reward system for active skiers and mountaineers in 1966. He established the ‘Grand Tour’, a round trip between all AAC lodges.^202^ Every participant received a specially prepared ‘passport’ to be marked at each lodge as well as a map showing the location of all huts. After visiting all lodges, one was entitled to be rewarded with a ‘special badge’.^203^ Such reward systems were already well-known and widely used by Austrian tourism associations to encourage sportsmanship.

During that time, safety issues had become increasingly essential in attracting ski tourists to the mountains. ‘All across the world, thickening networks of lifts and trails colored by difficulty suggested that the sport was fun and safe for the whole family’, as historian Andrew Denning described it.^204^ Inspired by international developments, Anton and Tommy Tomasi, another refugee from Europe, established a system of professional ski patrolmen supplied with Austrian safety equipment,^205^ which played a ‘pioneering role in mountain safety’.^206^

As a next step, Anton turned towards Mount Hotham in Victoria that offered good opportunities to develop a ‘Thredbo-like’ ski resort.^207^ He prepared a report with specific recommendations concluding that ‘the region has limitless possibilities and an infinite variety of slopes second to none in Australia’.^208^ Anton again gathered a syndicate around him, aimed at ‘making Hotham a second Thredbo’.^209^ However, the venture finally failed, because there was no access road to the foot of the ski runs and the cost of building one was far beyond the Syndicates’ means.^210^ His next step was to establish an AAC lodge in the Victorian ski resort of Mount Buller. In 1964, AAC issued invitations to well-known Buller skiers. At that time, the Winter Olympics took place in Innsbruck and the thoughts of skiers around the world were fixed on Patscherkofel Mountain near Innsbruck. After securing a lease for the project, AAC decided to name the lodge after the famous Austrian Mountain. When ‘Patscherkofel Lodge’ officially opened in May 1966, Anton invited various celebrities, such as then Prime Minister Harold Holt, to ‘assist the sale of lodge memberships’.^211^ Anton and his son Phillip attended the opening ceremony ‘in traditional Austrian *Lederhosen* [leather pants], embroidered weskits and feathery Tyrolean hats’.^212^

‘Patscherkofel lodge’ was Anton’s last project. He died on 17 September 1966 of meningococcal septicemia. After his death, the *Canberra Times* recalled: ‘Some of the colour has gone from the Australian snow scene’.^213^ One year after his death, the Minister of Land, Thomas Lewis, announced that a so-far unnamed peak near Mount Kosciusko would be named ‘Mount Anton’, ‘because of the substantial contribution of Mr. Anton to advance Australian skiing’.^214^ In June 1968, Anton again was at the centre of a major Thredbo event, when the ski season was opened by a church service conducted in his memory. At that occasion, his urn was buried in a rock at Mount Crackenback close to Thredbo.^215^

## Conclusion

This paper has provided an insight into the pioneering phase of Australian post-war skiing. It focused on Charles Anton’s activities as a ‘social agent’ and showed how he accumulated, transferred, and, finally, adapted knowledge gained in Austria to the needs of skiers in Australia, eventually creating one of the largest ski clubs as well as the most international ski resort in Australia. During his years in Austria, Anton accumulated knowledge of the working structures of the Austrian Alpenverein that later in Australia proved to be of great use for him. He came to a country that was very much underdeveloped in terms of skiing infrastructure. After recognizing a great demand for ski accommodation in the Snowy Mountains, he successfully established an organization based on his knowledge of the Austrian Alpenverein. It had a very inclusive structure and was open to all mountaineers which distinguished it from most of the existing ‘exclusive’ Australian ski clubs.

When coming to Australia, Anton was influenced by an Austrian interwar mountaineer attitude of rejecting mass tourism. In the start-up phase of Australian post-war skiing, his initial idea was very successful. However, after the nature of skiing changed and turned from a mere leisure activity of a few mountaineering well-offs into a ‘mass industry’, Anton had to adapt his plans in order to succeed. Consequently, he transformed the knowledge he had brought into his new home to the needs of a changed market. Ever more Australians were able to afford winter holidays during the post-war years and Anton transformed the idea of a club that aims at opening up the mountains through a dense network of shelter huts for bred-in-the-bone mountaineers into an organization that provides accommodation lodges in easily accessible ski resorts. Thus, the nature of his organization and his clientele changed, setting the stage for a period of quick expansion during the early 1960s.

Anton always kept himself well informed about skiing and observed trends and developments around the world. He maintained contacts with skiers and ski organizations in Austria and Australia, supporting an exchange of equipment, staff, and ideas between both countries. In response to new trends, he was one of the first to be involved in the formation of Australia’s ski resorts as early as the late 1950s. By that time, this paper has shown, he had revized his initial rejection of mass tourism and had focused on commercial projects.

A further interesting aspect of his life was the strong contradiction between his pride in being assimilated, and his embrace of Austrian cultural paraphernalia. Austrian elements were ubiquitous in his private as well as his business life. This paper argues that Anton, who was always keen to stand out from the others, successfully exploited Austria’s high status among Australian skiers to get publicity for his projects as well as to advertize skiing in general. It showed that sentimentality towards his old homeland did not play an important role for him. He rather used Austrian elements for marketing purposes and to attract attention. Anton knew about the importance of a dense network of supporters. He never hesitated to contact high-ranking persons and was always able to ‘find the right person for the right job at the right time’, as one of his business partners described. He left a lasting impression on Australia’s ski landscape: By the time of his death, he had built up the largest ski and alpine club in the country with more than 1,000 members and six lodges.^216^

## Disclosure Statement

No potential conflict of interest was reported by the author.

## Funding

This work was supported by the Austrian Science Fund (FWF) [J-3744] and the Austrian Zukunftsfonds [P16-2403].

## Notes on Contributor

***Philipp Strobl***, PhD, is an Erwin Schrödinger Fellow of the Austrian Science Fund (FWF) and a researcher at Swinburne University of Technology, Melbourne (Australia). His research is focused on the history of migration, globalization, and urban history.

## Notes

1. ‘New Alpine Cabin to Be Highest House in Australia’, *Sydney Morning Herald*, 8 December 1950, 2.

2. Patrick Thorne, ‘Fifty Years of Skiing History’, *Independent*, 15 October 2011, http://www.independent.co.uk/travel/skiing/fifty-years-of-skiing-history-2370714.html (accessed 18 July 2016).

3. A wide variety of pre- and post-war migrants from Central Europe had used their alpine experience to advance skiing in Australia: Austrian, the Czech, Italian, Swiss, German, and Hungarian migrants such as Eddy Hausegger, Helmut Kofler, Tommy Tomasi, Rudi Würth, Hans Grimus, Tony Sponar, Sasha and Karel Nekvapil, Max Bruck, Ernst and Aurel Forras, Tony Mathies, Garry Fischer, Charles William Anton, and many more had either developed skiing infrastructure (hotels, lodges, ski lifts, ski patrols), or had improved ski instruction thus leaving a deep mark on the Australian skiing community.

4. E. John and B. Allen, *Historical Dictionary of Skiing* (Lanham: Scarecrow Press, 2012), ix.

5. Annette R. Hofmann, ‘Introduction’, *The International Journal of the History of Sport* 30, no. 6 (2013), 561.

6. The probably most acknowledged is E. John B. Allen, who offers a broad international approach: E. John and B. Allen, *The Culture and Sport of Skiing: From Antiquity to World War II* (Amherst: University of Massachusetts Press, 2007); Allen, *Historical Dictionary*; Andrew Denning provides a multinational overview of certain aspects of skiing such as cultural or environmental changes: Andrew Denning, *Skiing into Modernity: A Cultural and Environmental History* (Berkeley: University of California Press, 2015); A special issue of *The International Journal of the History of Sport* published in 2013 deals with a wide range of case studies showing the diversity of skiing in Europe: Hofmann, ‘Introduction’, 561, 562.

7. Wendy Cross, *Australian Skiing: The First 100 Years* (Sydney: Walla Walla Press, 2012).

8. Peter Southwell-Keely, *Highway to Heaven. A History of Perisher and the Ski Resorts Along the Kosciuszko Road* (Gordon: NSW Perisher Historical Society, 2013); and Peter Southwell-Keely, *Out on the Tops. The Centenary of the Kosciuszko Alpine Club* (Gordon: NSW Perisher Historical Society, 2009).

9. Mitchell G. Ash, ‘Wissens- und Wissenschaftstransfer: Einführende Bemerkungen’, *Berichte zur Wissenschaftsgeschichte* 29 (2006), 181–9.

10. Johannes Paulmann, ‘Internationaler Vergleich und Interkultureller Transfer. Zwei Forschungsansätze zur europäischen Geschichte des 18. Und 19. Jahrhunderts’, *Historische Zeitschrift* 267 (1998), 649–85.

11. Veronika Lipphardt and David Ludwig, ‘Knowledge Transfer and Science Transfer’, *European History Online* (2011), http://www.ieg-ego.eu/lipphardtvludwigd-2011-en (accessed 16 June 2016).

12. Paulmann, ‘Internationaler Vergleich’, 676.

13. Ibid., 674.

14. Ibid., 680.

15. Stefan Manz and Panikos Panayi, ‘Refugees and Cultural Transfer to Britain: An Introduction’, *Immigrants & Minorities* 30, nos 2–3 (2012), 133.

16. National Archives of Australia (NAA), SCHWARZ Alois, A997, 1938/662.

17. Harriet Pass Freidenreich, *Jewish Politics in Vienna 1918*–*1938* (Bloomington: Indiana University Press, 1991), 5.

18. See note 16.

19. National Archives of Australia (NAA), Stella Schwarz and Charles William Anton, NSW, A12217, L1.

20. National Archives of Australia (NAA), Anton Charles William, B883, NX181034.

21. See note 19.

22. Leon Smith (friend of Anton’s and his successor as president of the AAC), in discussion with the author, Sydney, March 2016.

23. ‘Veraenderungen bei Unternehmungen’, *Neues Wiener Tagblatt*, 27 May 1938, 13.

24. Tony Sponar, *Snow in Australia?* – *That’s News to Me* (Palmerston: Tabletop Press, 1995), 24.

25. Rudolf Müllner, ‘The Importance of Skiing in Austria’, *The International Journal of the History of Sport* 30, no. 6 (2013), 659–73.

26. Anneliese Gidl, *Alpenverein: Die Städter entdecken die Alpen* (Vienna: Böhlau, 2007), 113.

27. Ibid., 83.

28. Ibid., 249.

29. Ibid., 147.

30. Ibid., 147.

31. Sponar, *Snow in Australia*, 24.

32. Ibid., 24.

33. Lee Wallace Holt, ‘Mountains, Mountaineering and Modernity: A Cultural History of German and Austrian Mountaineering, 1900–1945’ (PhD diss., University of Texas, 2008), 52.

34. Andrew Denning, ‘Alpine Skiing’, in John Nauright et al. (eds), *Sports Around the World: History, Culture, and Practice: Volume I* (Santa Barbara: ABC-Clio, 2012), 430–4.

35. Holt, ‘Mountains, Mountaineering and Modernity’, 68.

36. Ibid.

37. Ibid., 70.

38. For more information, see the section: ‘New Times Arriving!’ – Club lodges and Thredbo.

39. Robert Paxton, *The Anatomy of Fascism* (New York: Alfred A. Knopf, 2004), 115.

40. For more information, see: Guenter Bischof, Anton Pelinka, Alexander Lassner (eds), *The Dollfuss/Schuschnigg Era in Austria: A Reassessment* (New Brunswick: Transaction Publisher, 2003).

41. NAA, Stella Schwarz and Charles William Anton, A12217, L1.

42. Helfried Pfeifer, *Die Ostmark. Eingliederung und Neugestaltung. Historisch*-*systematische Gesetzessammlung nach dem Stande vom 16. April 1941* (Vienna: Staatsdruckerei, 1941), 24.

43. Richard Chesnoff, *Pack of Thieves: How Hitler and Europe Plundered the Jews and Committed the Greatest Theft in History* (New York: Doubleday, 1999), 24.

44. Hannelore Burger, *Heimatrecht und Staatsbürgerschaft österreichischer Juden: vom Ende des 18. Jahrhunderts bis in die Gegenwart* (Vienna: Böhlau, 2014), 148.

45. See note 20.

46. Ibid.

47. Burger, *Heimatrecht und Staatsbürgerschaft österreichischer Juden*, 148.

48. See note 31.

49. See note 22.

50. National Archives of Australia (NAA), Charles William ANTON, SP11/15, Anton Charles William.

51. National Archives of Australia (NAA), ANTON Charles William, A435, 1944/4/1110.

52. See note 50.

53. Ibid.

54. Warren Peck (friend of Anton and former president of the AAC), in discussion with the author, Melbourne, June 2016.

55. See note 22.

56. Karl Bittmann, ‘Adapting to a New Land. Australia’, in Karl Bittmann (ed.), *Strauss to Mathilda: Viennese in Australia 1938*–*1988* (Leichhardt: Wenkart Foundation, 1988), 16.

57. National Archives of Australia (NAA), Stella Schwarz and Charles William, A12217, L1.

58. See note 45.

59. Kerry Regan, ‘Anton, Charles William (1916–1966)’, *Australian Dictionary of Biography*, National Centre of Biography, Australian National University, http://adb.anu.edu.au/biography/anton-charles-william-9372/text16463 (accessed 17 May 2016).

60. Charles William Anton, ‘Alien Labour Companies’, *Sydney Morning Herald*, 1 September 1942, 3.

61. Klaus Neumann, *Across the Seas: Australia’s Response to Refugees: A History* (Collingwood: Black Ink, 2015), 71.

62. Neumann, *Across the Seas*, 73.

63. See note 52.

64. See note 45.

65. See note 52.

66. See note 45.

67. Cross, *Australian Skiing*, 3.

68. Ibid., 17.

69. Ibid., 21.

70. For a description of the early ski clubs, see: ibid., 19ff.

71. Southwell-Keely, *Out on the Tops*, 109.

72. Charles Anton, *Brief History and Aims of the Australian Alpine Club (Incorporating Ski Tourer’s Association of Australia)*, Brochure, February 1963.

73. See note 45.

74. Cross, *Australian Skiing,* 31.

75. Anton, *Aims of the Australian Alpine Club*.

76. See note 74.

77. Regan, ‘Anton, Charles William.’

78. See note 22.

79. Ibid.

80. Jill MacDonald, ‘Our Backward state’, *Australian Ski Year Book* (1950), 44; and ‘We Have Neglected Our Snowfields’, *Sunday Herald*, 28 September 1952, 7.

81. See note 75.

82. Wendy Cross, *Twenty*-*One Years of the Australian Alpine Club* (Melbourne: Massina Print, 1972), 26.

83. Jim Darby, *Thredbo 50. 1957 to 2007* (Mount Macedon: TSM Publishing, 2006), 12.

84. Don Richardson, ‘The Lake Albina Project’, *Australian Ski Yearbook* (1950), 37.

85. Ibid., 38.

86. See note 75.

87. Leon Smith, ‘Charles W. Anton’, (Unpublished essay, Sydney, 1986).

88. See note 22.

89. Cross, *Twenty*-*One Years of the Australian Alpine Club*, 4.

90. See note 1.

91. William Hudson, ‘The Snowy Mountains Scheme’, *Nature* 195 (1962), 16.

92. Sponar, *Snow in Australia*, 21.

93. Margaret Anton, ‘Skiers Build Australia’s Highest Habitation’, *Australian Women’s Weekly*, 14 April 1951, 26.

94. Bob Arnott, ‘A Hut at Lake Albina’, *SKI*-*Horizon* (1950), 18.

95. See note 1.

96. It was the authority responsible for electricity supplies in Sydney.

97. ‘Lake Albina Ski Lodge’, *Sydney Morning Herald*, 17 May 1951, 11.

98. Smith, ‘Charles W. Anton’.

99. Margaret Anton and Charles William Anton, ‘Drift Skiing in Mid-Summer’, *Australian Ski Yearbook* (1949), 57.

100. Ibid., 57.

101. Charles Anton, ‘Summer Slalom’, *Australian Ski Yearbook* (1952), 57.

102. Cross, *Twenty*-*One Years of the Australian Alpine Club*, 12.

103. See note 98.

104. Cross, *Twenty*-*One Years of the Australian Alpine Club*, 13.

105. Charles Anton, ‘STA-Report’, in *Australian Skiing Yearbook* (1954), 82.

106. Cross, *Twenty*-*One Years of the Australian Alpine Club*, 44.

107. See note 98.

108. Warren Peck, ‘In Memoriam Charles Anton’ (Unpublished eulogy, Melbourne, 1966).

109. Peck, ‘In Memoriam Charles Anton’.

110. Elyne Mitchell, *Discoverers of the Snowy Mountains* (South Melbourne: Macmillan, 1985), 123.

111. Smith, ‘Charles W. Anton’.

112. ‘Tyrolean Ski Hut at Kosciusko’, *Australian Women’s Weekly*, 9 September 1953, 23.

113. Cross, *Australian Skiing*, 149.

114. Cross, *Australian Skiing*, 142.

115. Darby, *Thredbo*, 84.

116. See note 75.

117. ‘Tyrolean Ski Hut’, 23.

118. ‘Skiing Clubs Ready for Kosciusko’, *Sunday Herald*, 22 February 1953, 22.

119. See note 74.

120. Southwell-Keely, *Out on the Tops*, 101.

121. Philipp Strobl, *Innsbrucker Wirtschaftsgeschichte* (Innsbruck: Studienverlag, 2013), 114.

122. Ibid., 115.

123. R.M. Simmers, ‘Ski Tow Data’, *Australian Ski Yearbook* (1950), 22.

124. ‘Highest Ski Tow is Opened’, *Sunday Herald*, 13 July 1954, 9.

125. Charles William Anton, Northcote Ski Tow Project, letter to the members of the STA, 5 December 1952.

126. See note 124.

127. Charles Anton, ‘Ski Tourers’ Association’, *Australian Ski Yearbook* (1955), 71.

128. Peck, ‘In Memoriam Charles Anton.’

129. See note 111.

130. ‘Young Couple Furnish Flat as Chalet’, *Australian Women’s Weekly*, 23 September 1950, 44, 45.

131. Ibid., 44, 45.

132. See note 111.

133. Smith, ‘Charles W. Anton;’ and Peck, ‘In Memoriam Charles Anton.’

134. Cross, *Australian Skiing*, 32.

135. See note 130.

136. ‘Ready for Kosciusko’, 22.

137. See note 75.

138. See note 74.

139. See note 75.

140. See note 74.

141. See note 31.

142. Denning, ‘Alpine Skiing’, 434.

143. Andrew Denning, ‘How Skiing went from the Alps to the Masses’, *Atlantic*, 23 February 2015, http://www.theatlantic.com/business/archive/2015/02/how-skiing-went-from-the-alps-to-the-masses/385,691/ (accessed 1 August 2016).

144. Denning, ‘Alpine Skiing’, 432.

145. David Scott, ‘Ski Lodges on the Summit – A History of the Ski Tourers’ Association Sites’, Kosciusko Huts Association, http://www.khuts.org/images/stories/history/LodgesOnTheSummit_dscott_aug2013.pdf (accessed 15 June 2016).

146. Ibid.

147. ‘Lifesavers – In the Snow’, *Australian Women’s Weekly*, 23 August 1961, 5.

148. Sponar, *Snow in Australia*, 150.

149. Ibid., 134.

150. Southwell-Keely, *Out on the Tops*, 125.

151. See note 75.

152. Sponar, *Snow in Australia*, 134.

153. Ibid., 134.

154. Geoffrey Hughes, *Starting Thredbo* (Walarah: G.E.F. Hughes, 2008), 18.

155. Sponar, *Snow in Australia*, 142.

156. Southwell-Keely, *Out on the Tops*, 129.

157. Ibid., 129.

158. See note 155.

159. ‘Alpine Hotel and Chairlift for Thredbo Valley’, *Canberra Times*, 10 February 1958, 7.

160. Cross, *Twenty*-*One Years of the Australian Skiing*, 74.

161. See note 159.

162. See note 16.

163. See note 155.

164. See note 75.

165. Ibid.

166. Charles Anton, ‘Kareela Hutte’, in *Australian Ski Yearbook* (1959), 98.

167. See note 75.

168. ‘News Items’, *Australian Ski Yearbook* (1958), 66.

169. See note 16.

170. Ibid., 74.

171. See note 111.

172. Cross, *Twenty*-*One Years of the Australian Alpine Club*, 21.

173. Darby, *Thredbo*, 78.

174. Sponar, *Snow in Australia*, 143.

175. Hughes, *Thredbo*, 27.

176. Southwell-Keely, *Out on the Tops*, 131.

177. Cross, *Australian Skiing*, 76; and Darby, *Thredbo*, 16.

178. Hughes, *Thredbo*, 41.

179. See note 128.

180. ‘Offer for Thredbo Company Accepted’, *Canberra Times*, 10 November 1961, 26.

181. See note 77.

182. See note 111.

183. Ibid.

184. See note 128.

185. See note 75.

186. Ibid.

187. Warren Peck, ‘Part 8.2 – The Alps at the Crossroads and the AAC beyond 1962’, Australian Alpine Club, http://www.australianalpineclub.com/heritage/81-part-8-2-the-alps-at-the-crossroads-and-the-aac-beyond-1962.html?showall=1 (accessed 20 June 2016).

188. Ibid.

189. Ibid.

190. See note 75.

191. See note 109.

192. Cross, *Twenty*-*One Years of the Australian Alpine Club,* 26.

193. See note 77.

194. See note 111.

195. Denning, ‘From the Alps to the Masses’.

196. See note 75.

197. Ibid.

198. See note 180.

199. Peck, ‘The Alps at the Crossroads’.

200. ‘Austrian Visit Certain’, *Canberra Times*, 23 June 1966, 29.

201. See note 111.

202. Cross, *Twenty*-*One Years of the Australian Alpine Club*, 30.

203. Ibid., 30.

204. See note 204.

205. ‘Lifesavers – In the Snow’, *Australian Women’s Weekly*, 23 August 1961, 5; and Darby, *Thredbo*, 81.

206. Ibid., 83.

207. See note 109.

208. See note 75.

209. See note 109.

210. Ibid.

211. See note 199.

212. Ibid.

213. ‘Orbituary: A Pioneer of the Ski Scene’, *Canberra Times*, 20 September 1966, 4.

214. ‘Peak in Snow Honours Skier’, *Canberra Times*, 23 September 1967, 37.

215. ‘Thredbo Packed for ski Opening’, *Canberra Times*, 10 June 1968, 3.

216. See note 109.

